# How Efficient Is My (Medicinal) Chemistry?

**DOI:** 10.3390/ph9020026

**Published:** 2016-05-16

**Authors:** Jean Jacques Vanden Eynde

**Affiliations:** Laboratory of Organic Chemistry, University of Mons-UMONS, Place du Parc 23, Mons B-7000, Belgium; jean-jacques.vandeneynde@ex.umons.ac.be; Tel.: + 32-2-355-8161

**Keywords:** atom economy, ecoscale, E factor, green chemistry, life cycle analysis, paracetamol, reaction mass efficiency, stoichiometric factor, waste, yield

## Abstract

“Greening” a chemical transformation is not about only changing the nature of a solvent or decreasing the reaction temperature. There are metrics enabling a critical quantification of the efficiency of an experimental protocol. Some of them are applied to different sequences for the preparation of paracetamol in order to understand their performance parameters and elucidate pathways for improvement.

## 1. Introduction

As largely emphasized during the recent United Nations Conference on Climate Change (COP21 [1]) held in Paris (France), nowadays there is an increasing awareness and urgent necessity of limiting, as far as possible, any source of pollution and accumulation of waste. Consequently, the environmental legislation becomes ever more severe in order to respond to the public pressure and to the demands of green movements. On the other hand, economical constraints lead chemical industries to improve their processes, to reduce the cost of their production, and to accelerate the discovery of novel commercial products. Faced with that situation, scientists have to explore new ways to perform chemical transformations and the disclosure of innovative experimental procedures emerges as a fascinating and inescapable challenge. In that sense, the past few years have been particularly fruitful with the intensive use of supported reagents as well as with the introduction of ultrasound generators and microwave ovens in research laboratories. Pharmaceutical companies have benefited in addition from the development of combinatorial chemistry, which has been accompanied by an exploding renewal of interest for solid phase synthesis. Besides those methodologies, a novel concept arose at the end of the 20th century under the name of *Green Chemistry* [2]. In many papers you can read that environmentally friendly protocols have been developed to improve known reactions or to access original compounds. However, are those protocols as green as claimed? Do they really follow the principles of *Green Chemistry*? Are they characterized by favorable metrics, having in mind that any amount of a substance that is not incorporated in the final product will become a waste if it cannot directly be reused? This year, the ACS Green Chemistry Institute will organize, in Portland (OR, USA), the 20th Annual Green Chemistry and Engineering Conference [3]. This is a pretext and an opportunity to remind us, in this review, of some now familiar but useful tools for a critical evaluation of the performance and environmental impact of a chemical reaction.

## 2. The Twelve Principles of Green Chemistry and the Principles of Green Engineering

**Figure 1:**
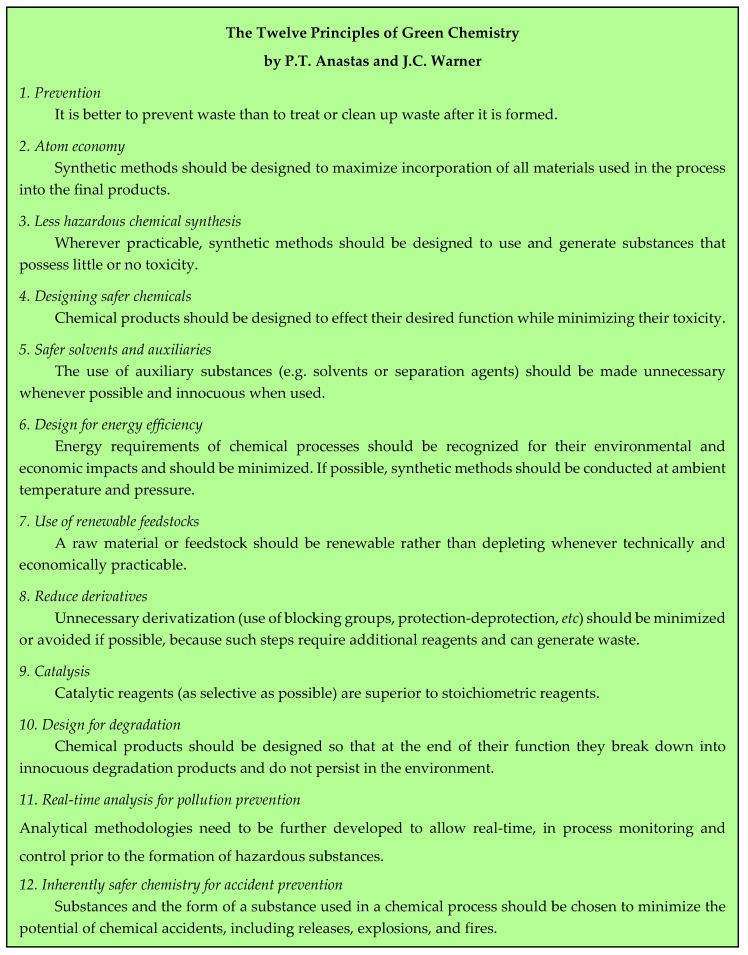
The twelve principles of Green Chemistry [2].

**Figure 2:**
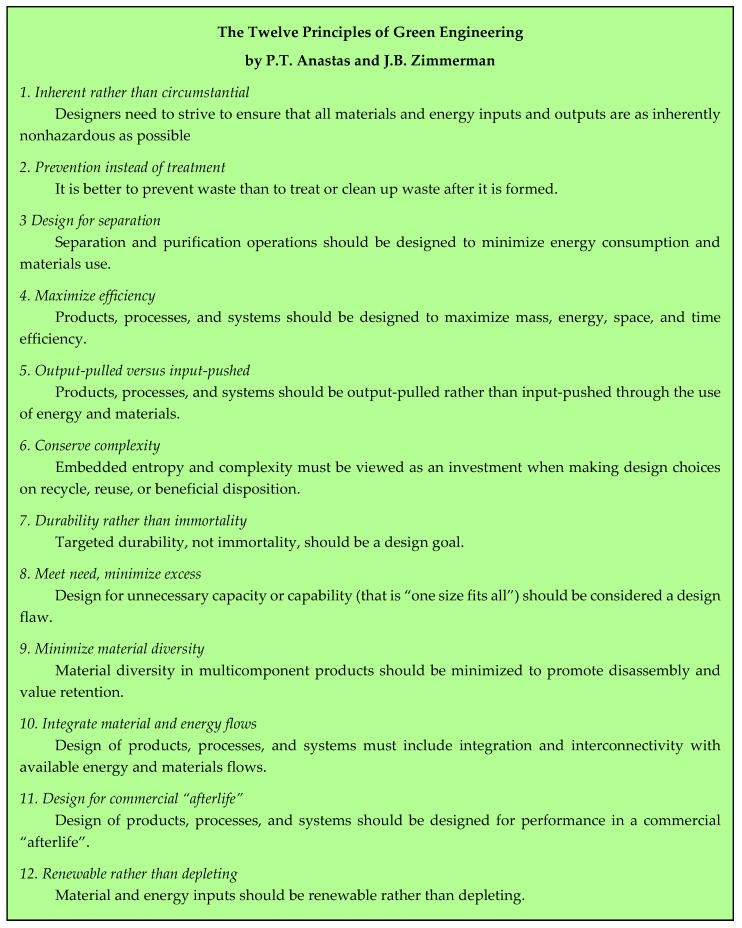
The twelve principles of Green Engineering [4].

**Figure 3:**
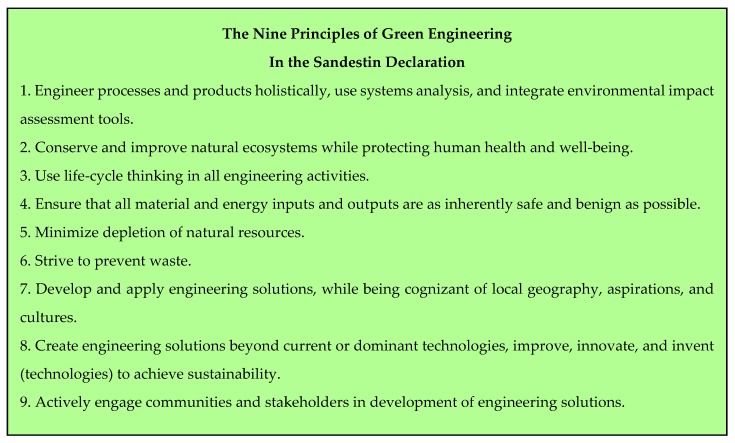
The Sandestin declaration [3].

The publication of the Twelve Principles of Green Chemistry by Anastas and Warner in 1998 [2] has undoubtedly been the most efficient tool for the development of chemical procedures aiming to limit the amount of waste and to protect the environment. Extrapolation of those concepts to the industrial world can be found in the Twelve Principles of Green Engineering, disclosed in 2003 by Anastas and Zimmerman [4] and in the Nine Principles of Green Engineering, which are described in the Sandestin Declaration [5]. The three lists are reported in Figure 1, Figure 2 and Figure 3. Mention could also be made of twelve more principles of Green Chemistry added in 2001 by Winterton [6] and Six Principles of Sustainability established by engineering institutions in the United Kingdom [7].

## 3. Some Synthetic Routes to Paracetamol

**Figure 4:**
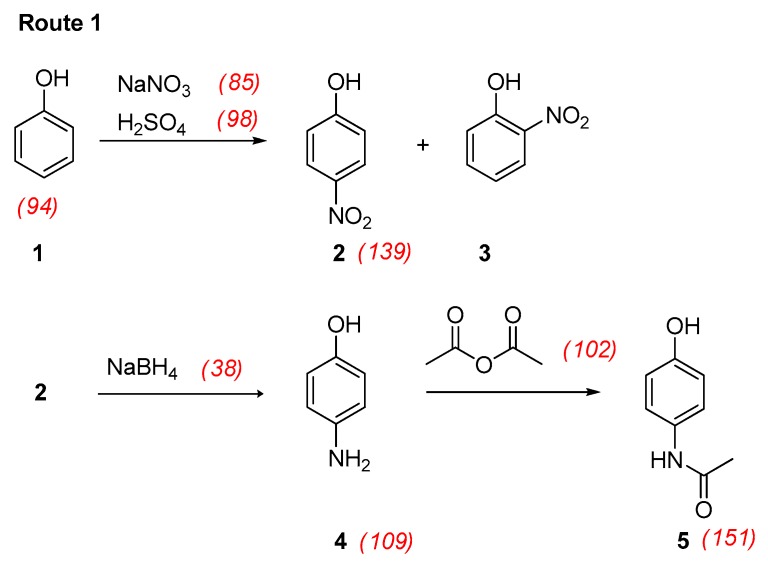

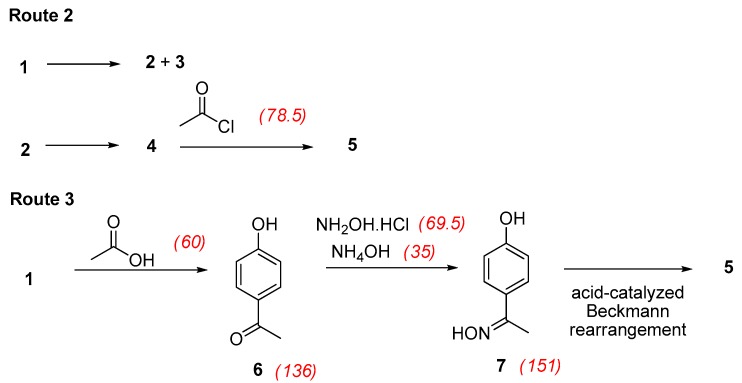
Some synthetic routes for the preparation of paracetamol (molecular weights of reactants and products are indicated in red and in brackets).

Paracetamol (acetaminophen, *N*-(4-hydroxyphenyl)acetamide, **5** in Figure 4) is a well-known analgesic that can be prepared by different synthetic routes from phenol (**1**). The most usual protocol [8,9,10,11] starts by a nitration reaction (Figure 4). After separation of the *para* isomer from the *ortho* isomer, 4-nitrophenol (**2**) is reduced into 4-aminophenol (**4**). The latter is finally *N*-acetylated, most often by treatment with acetic anhydride (Route 1) but sometimes using acetyl chloride (Route 2). Hoechst-Celanese [12,13] has proposed a quite different approach (Route 3). It involves acetylation of phenol by action of acetic acid catalyzed by HF. Reaction of 4′-hydroxyacetophenone (**6**) with hydroxylamine yields the corresponding oxime (**7**) that is converted into paracetamol by an acid-catalyzed Beckmann rearrangement.

The following experimental procedures will be considered in this review. They have been adapted so that each route starts from 100 mmol of phenol and the total amount of an isolated product is systematically engaged in the subsequent step. A particular attention is dedicated to the *N*-acetylation of 4-aminophenol because different protocols have been reported in the literature, thus enabling a comparison of their respective efficiencies.

### 3.1. Nitration of Phenol

Place a three-necked round bottom flask in an ice water bath and place a thermometer into one of the necks. Place 15.00 g of sodium nitrate into the flask, add 40 mL of water and stir. Cautiously add concentrated sulfuric acid (13.6 mL; 25.00 g) to the stirred solution. Slowly add solid phenol (9.40 g, 100 mmol) at such a rate that the temperature of the solution does not rise above 20 °C (about a half a spatula at a time over about 20 min) and then stir, preferably with a magnetic stirrer, for 2 h. Remove the thermometer. Decant off the supernatant liquid and add water (25–30 mL) to the residue. Put a dropping funnel into one of the necks and a stillhead and condenser into another. Insert a stopper in the third. Heat the mixture, and distil off one of the components with the steam. At the same time, add water to the mixture through the dropping funnel at a similar rate. Stop the distillation once the product has stopped coming over, and then filter the distillate to give crystals of the 2-nitrophenol isomer (4.17 g; 30 mmol) in 30% yield following [9]. Cool the residual solution in the distillation flask, then filter off the other solid isomer (4-nitrophenol; 5.14 g; 37 mmol) in 37% yield following [9] and recrystallize it from 0.5 M hydrochloric acid [8].

### 3.2. Reduction of 4-Nitrophenol

Place 51.4 mL (51.4 mmol) of 1 M sodium hydroxide in a conical flask. Add 2.88 g (76 mmol) of sodium borohydride, followed by 50 mg of palladium on charcoal (5% or 10%). Cool in ice to approximately 13 °C. Add 5.14 g (37 mmol) of 4-nitrophenol in very small portions (half a microspatula at a time) over 30 min. Make sure the temperature is kept between 13–17 °C during the addition. After the addition is complete, the mixture should be stirred for a further 15 min and acidified with 2 M hydrochloric acid (about 87 mL). Filter the mixture to remove catalyst and adjust the filtrate to pH 7–8 by carefully adding solid sodium hydrogenocarbonate a little at a time. Filter off the precipitate and wash with a little cold water to give 4-aminophenol (2.98 g; 27 mmol; 74%) after drying [8].

### 3.3. Acetylation of 4-Aminophenol

#### 3.3.1. With Diluted Acetic Anhydride

Place 2.98 g (27 mmol) of 4-aminophenol and 27 mL of distilled water in a 100 mL conical flask and stir briskly at room temperature in order to suspend the solid in the water. In a fume cupboard, add 3.49 g (34 mmol) of acetic anhydride to the stirred suspension and gently shake to mix. The solid will dissolve after about 30 s. Continue shaking and a precipitate will form after 2 min. After 10 min, the solid should be filtered off under suction, washed with a little cold water and dried (2.47 g; 16 mmol; 60%). The product may be purified by crystallization from distilled water [8].

#### 3.3.2. With Pure Acetic Anhydride in the Presence of a Catalyst

Mix acetic anhydride (7.4 mL, 7.99 g, 78 mmol), 4-aminophenol (2.98 g, 27 mmol), and H_14_[NaP_5_W_30_O_110_] (0.02 g) and stir at room temperature for 15 minutes. At the end of the reaction, dilute the mixture with water (74 mL) and the crude product precipitates by cooling in an ice bath. Yield: 91% (3.77 g, 25 mmol) [10].

#### 3.3.3. With Acetic Anhydride under Solvent-Free and Catalyst-Free Conditions

Transfer 27 mmol (2.98 g) of 98% pure 4-aminophenol and 27 mmol (2.79 g) of 99% pure acetic anhydride to a rotary stainless steel ball mill equipped with an exhaust and a scrubber system. Add a slight excess amount (0.03 g) of acetic anhydride to this mixture. First mix gently and then grind the mixture to about 10 μm particle size. Yield: 3.99 g, 26 mmol, 97% [11].

#### 3.3.4. With Acetic Chloride under Solvent-Free and Catalyst-Free Conditions

Transfer 27 mmol (2.98 g) of 98% pure 4-aminophenol and 27 mmol (2.14 g) of 99% pure acetyl chloride to a rotary stainless steel ball mill equipped with an exhaust and a scrubber system. Add a slight excess amount (0.03 g) of acetyl chloride to this mixture. First mix gently then grind the mixture to about 10 μm particle size. Yield: 4.10 g, 26 mmoles, 99% [11].

### 3.4. Acetylation of Phenol

Charge phenol (9.40 g, 100 mmol) and acetic acid (12.00 g, 200 mol) to a 300 mL Hastelloy C autoclave at room temperature. Evacuate and cool the reactor to −20 °C. HF (100 g, 5 mol) and then transfer it into the reactor. Heat the reactor to 80 °C and maintain for 1 h at reaction temperature. At the end of the reaction, cool the reactor to 20 °C and vent the excess HF to a KOH scrubber. Add Ethyl acetate to the contents of the reactor. Neutralize the mixture with 45% aqueous KOH. Separate the resulting organic phase, dry it over MgSO_4_ and allow it to evaporate to afford a yellow solid that contains 13.10 g (96 mmol, 96%) of 4′-hydroxyacetophenone [12].

### 3.5. Formation of the Oxime of 4′-Hydroxyacetophenone

Prepar a solution by adding 13.10 g (96 mmol) of 4′-hydroxyacetophenone, 7.30 g (105 mmol) of hydroxylamine hydrochloride, and 9.60 g of water to 38.5 mL of ethanol. To the solution, add 4.80 g of 30% ammonium hydroxide, and then heat at reflux for 2 h. Remove the ethanol in a rotary evaporator to yield a yellow oil. The extractive work-up affords 14.50 g (96 mmol, 99%) of 4′-hydroxyacetophenone oxime [12].

### 3.6. Formation of Paracetamol by an Acid-Catalyzed Beckmann Rearrangement

Heat a solution of 14.50 g (96 mmol) of 4′-hydroxyacetophenone oxime and 109.10 g of trifluoroacetic acid at reflux under a nitrogen atmosphere. Remove the trifluoroacetic acid in a rotary evaporator to afford 21.40 g of oil, and then dissolve the oil in 100 mL of water. Cool to 0 °C for 0.5 h, until crystallization occurs. Filtrate and dry the crystals to yield 10.30 g (68 mmol, 71%) of paracetamol [12].

## 4. Mass Balances

When we perform a chemical reaction, we have to follow a chemical equation and it will immediately indicate the minimum amount of waste we can expect from the experimental process. Indeed, any material that will not enter in the composition of the final product will constitute a waste in one way or another. That concerns reactants and product(s) only. Some associated metrics are atom economy, yield, reaction mass efficiency as defined by Curzons, and stoichiometric factor. In a further step of the evaluation of the environmental impact of a chemical transformation, solvents and any substance used as auxiliary or in the work-up procedure can also be considered. In that case, global reaction efficiency, effective mass yield, process mass intensity, and E-factor will be considered.

### 4.1. When Reactants and Product(s) Only Are Considered

#### 4.1.1. Atom Economy: A Theoretical Value

Atom Economy (AE) is defined [14,15] as “the molecular weight of the isolated product divided by the sum of the molecular weights of the reactants”, the result being expressed in %:

AE = (MW_isolated product_/Σ MW_reactants_) × 100

In the case of preparation of paracetamol following Route 1, as described in Figure 4, we have:

AE = [151/(94 + 98 + 85 + 38 + 102)] × 100 = 36%

where 151 is the molecular weight of paracetamol and 94, 98, 85, 38, and 102 are the molecular weights of phenol, sulfuric acid, sodium nitrate, sodium borohydride, and acetic anhydride, respectively.

That means that in an ideal process, 64% of the atomic mass of the engaged reactants will constitute waste. This is a purely theoretical minimum value that cannot be modified except when we change the balanced chemical equations. Thus, by replacing acetic anhydride with acetyl chloride (molecular weight: 78.5) as the acetylating agent (Route 2), the atom economy is insignificantly increased by 2%:

AE = [151/(94 + 98 + 85 + 38 + 78.5)] × 100 = 38%

However, if the synthetic sequence is totally modified, as in the Hoechst-Celanese procedure (Route 3), then the atom economy rises to:

AE = [151/(94 + 60 + 69.5 + 40)] × 100 = 58%

where 94, 60, 69.5, and 40 represent the molecular weights of phenol, acetic acid, hydroxylamine hydrochloride, and sodium hydroxide, respectively, the other substances being used as catalysts. Catalysts are not present in the AE calculation because their atomic material is not consumed, but instead regenerated (at least theoretically), during the chemical process. The favorable increase of the atom economy in this procedure is associated with the facts that: (i) acetic acid (and not acetic anhydride, which has a higher molecular weight) is used for acetylation of phenol; and (ii) conversion of the oxime **7** into the amide **5** is a rearrangement reaction involving catalysts only. Addition and pericyclic reactions are other privileged chemical transformations contributing to a favorable atom economy.

Atom economy values calculated for protocols reported in Section 3.1, Section 3.2, Section 3.3, Section 3.4, Section 3.5 and Section 3.6 are gathered in Table 1.

#### 4.1.2. Yield: A Realistic Experimental Value

Yield is certainly one of the best known and most critical value associated with an individual chemical transformation. It reflects the efficiency of the process and thus the effective amount of waste created from the reactants. Yield, expressed in percent, is defined by:

Yield = [Obtained number of mole(s) of the desired product/Theoretical maximum number of mole(s) that can be obtained from the limiting reactant] × 100

Let us first consider the nitration of phenol (0.100 mol) as described in Section 3.1. 4-Nitrophenol (0.037 mol) was obtained in

Yield = (0.037/0.100) × 100 = 37%

That is a modest value, but it can be expected that unreacted reagents can be recovered and recycled in further runs. However, this will be possible only if those unreacted reagents were not consumed in unwanted transformations. At that point, the notions of selectivity and conversion are introduced. Following the concerned protocol, nitration of phenol is not a selective reaction because in addition to 4-nitrophenol, the compound expected and required for the synthesis of paracetamol, 2-nitrophenol was also obtained. Thus, from the starting 100 mmol of phenol, 67 mmol have been converted: 37 mmol were transformed into the expected product and 30 mmol were “lost” by formation of the *ortho* isomer. There are only 33 mmol of unreacted starting phenol left. They have not been recycled by the authors [8].

In a multistep synthesis, the overall yield is obtained by multiplying yields calculated for each individual step. For the preparation of paracetamol following Route 1 (Figure 4) and acetylation with diluted acetic anhydride (Section 3.3.1), paracetamol was obtained in an overall yield of:

Overall Yield = (0.37 × 0.74 × 0.60) × 100 = 16%

What is, in practice, considered as a (very) low yield. However, yields are values depending on the experimental conditions (and on the skills of the chemist!). That explains why various protocols (Section 3.3.1, Section 3.3.2 and Section 3.3.3) describing the *N*-acetylation step of 4-aminophenol with acetic anhydride furnished the final amide in different yields, as indicated in Table 1.

When Route 2 is considered (acetylation with acetyl chloride, Section 3.3.4), the overall yield slightly increases to:

Overall Yield = (0.37 × 0.74 × 0.99) × 100 = 27%

On the other hand, the Hoechst-Celanese synthetic pathway proposed in reference [12] emerges as a highly more favorable procedure because the overall yield rises to:

Overall Yield = (0.96 × 0.99 × 0.71) × 100 = 68%

#### 4.1.3. Reaction Mass Efficiency Following Curzons

Yields are calculated on the basis of the number of moles of the limiting reactant. In 2001, Curzons *et al.* [16], researchers at GlaxoSmithKline, defined the concept of “reaction mass efficiency” (RME_Curzons_) as the ratio between the isolated weight of the final product and the sum of the weights of the reactants:

RME_Curzons_ in % = (isolated weight of the desired product/sum of weights of the reactants) × 100

A value of 100 characterizes an ideal process.

For the nitration of phenol, we can calculate:

RME_Curzons_ = [5.14/(9.40 + 15 + 25)] × 100 = 10%

where 5.14 is the weight (in g) of 4-nitrophenol and 9.40, 15, and 25 are the weights (in g) of phenol, sodium nitrate, and sulfuric acid, respectively. The value means that 90% of the weights of the reactants used in the experiment are not inserted in the product (4-nitrophenol) and considered as waste in the protocol. This RME_Curzons_ value takes in account atom economy, yield, and stoichiometry [17].

In a multistep synthesis, the calculation of RME_Curzons_ requires the assumption that the entire amount of a product obtained in a reaction is engaged in the subsequent step. Therefore, we find for the overall preparation of paracetamol

- Following Route 1 and acetylation with diluted acetic anhydride
overall RME_Curzons_ = [2.47/(9.40 + 15 + 25 + 2.88 + 3.49)] × 100 = 4%
  where 2.47 is the weight (in g) of paracetamol and 9.40, 15, 25, 2.88, and 3.49 are the weights (in g) of phenol, sodium nitrate, sulfuric acid, sodium borohydride, and acetic anhydride, respectively;
- Following Route 2 (*N*-acetylation with acetyl chloride)
overall RME_Curzons_ = [4.10/(9.40 + 15 + 25 + 2.88 + 2.17)] × 100 = 8%
- Following Route 3 (Hoechst-Celanese process)
overall RME_Curzons_ = [10.33/(9.40 + 12.0 + 7.32 + 4.82)] × 100 = 31%

Comparison between the values associated with Routes 1–3 clearly demonstrates the superiority of the Hoechst-Celanese process in terms of RME_Curzons_. This is explained by the excellent overall yield and the absence of entering reagent in the rearrangement step.

RME_Curzons_ and overall RME_Curzons_ values calculated for protocols reported in Section 3.1, Section 3.2, Section 3.3, Section 3.4, Section 3.5 and Section 3.6 are gathered in Table 1.

#### 4.1.4. Stoichiometric Factor

The stoichiometric factor (SF) enables a comparison between an experimental process and a similar process in which all reactants should be used stoichiometrically [18]. Mention must be made that yield does not influence the value (in a similar way that it does not influence the value of the atom economy). The SF reflects the extent by which an excess of reagent(s) is used.

SF = 1 + [(sum of the weights of the reactants—sum of the weights of the reactant in a stoichiometric process)/sum of the weights of the reactant in a stoichiometric process].

For nitration of phenol, we have:

SF = 1 + {[(15.0 + 25.0 + 9.4) – (8.5 + 9.8 + 9.4)]/(8.5 + 9.8 + 9.4)} = 1 + 0.7 = 1.07

where (15.0 + 25.0 + 9.4) represents the sum of the weights (in g) of, respectively, sodium nitrate, sulfuric acid, and phenol used in the reaction and (8.5 + 9.8 + 9.4) the sum of the weights (in g) of the same reagents used in a stoichiometric manner (100 mmol). The value of 1.07 indicates that 7% of the weight of the reagents are used in excess in the reaction. There is no link between that value and atom economy, yield or amount of waste.

An overall stoichiometric factor [19] is calculated in a similar way considering the limiting reagent in the first step. Thus, for the preparation of paracetamol following Route 1 (Figure 4) and acetylation with diluted acetic anhydride (Section 3.3.1), we find:

SF = 1 + {[(15.0 + 25.0 + 9.4 + 7.75 + 12.75) − (8.5 + 9.8 + 9.4 + 3.8 + 10.2)]/(8.5 + 9.8 + 9.4 + 3.8 + 10.2) = 1 + 0.66 = 1.66.

In that overall sequence, 66% of the weights of the reagents were used in excess.

SF and overall SF values calculated for protocols reported in Section 3.1, Section 3.2, Section 3.3, Section 3.4, Section 3.5 and Section 3.6 are gathered in Table 1.

### 4.2. When Reactants, Product(s), As Well As Solvents and Any Additional Substance Are Considered

#### 4.2.1. Global Reaction Mass Efficiency and Effective Mass Yield

Global reaction mass efficiency (gRME) [19,20], seldom referred to as mass productivity (MP) [20], corresponds to a mass balance for the chemical process in its entirety. It is the ratio between the isolated weight of the final product and the sum of the weights of all materials entering in the reaction, including solvents, drying agents, washing solutions, and any other compound implemented during the experiment [21]:

gRME = isolated weight of the desired product/sum of weights introduced in the process

Generally, water is excluded from the calculation but it can be included; this remains a subject of controversies [21]. For nitration of phenol, when the weight of water is included, the value is

gRME = 5.14/(15 + 40 + 25 + 9.40 + 25 + 25) = 0.04

where 5.14 g represents the weight of 4-nitrophenol, 15, 25, and 9.40 g the weight of, respectively, sodium nitrate, sulfuric acid, and phenol; it is assumed that approximately 90 g of water is required in the protocol.

gRME and overall gRME values calculated for protocols reported in Section 3.1, Section 3.2, Section 3.3, Section 3.4, Section 3.5 and Section 3.6 are gathered in Table 1. From the data, it clearly appears that, except for the individual reactions of acetylation under solvent-free conditions (Section 3.3.3 and Section 3.3.4), all gRME values are below 10%. That means that more than 90% of the weight of the substances involved in the reactions were not incorporated in the final products and thus generated waste, if not recycled.

In green chemistry, the idea of effective mass yield (EMY) is also encountered [22]. The definition is similar to that of RME but only non-benign reactants are considered in the denominator. Many authors underline that the definition of non-benign material is not necessarily straightforward and some confusion can arise [23].

#### 4.2.2. Process Mass Intensity and E Factor

Process mass intensity (PMI) is calculated as the inverse of the global RME [15,18]. Thus:

PMI = 1/gRME

or

PMI = sum of the weights introduced in the process/isolated weight of the desired product.

Notice that RME varies from nearly 0 to 1 for an ideal process, whereas PMI ranges from 1 for an ideal process to higher values for less efficient cases. For the nitration of phenol, we find:

PMI =1/0.04 = 25

It is obvious that considering the amount of water in the process has a dramatic impact on the value. Indeed, when water is not taken into account, PMI drops to 9.6.

The E factor [24], where E stands for “environment”, is the ratio between the weights of everything used during the transformation but not incorporated in the desired product and the isolated weight of the desired product:

E factor = weight of waste/isolated weight of the desired product.

Process mass intensity (PMI) and E factor are linked by the relation:

PMI = E factor + 1

Following Sheldon [15], E factors (kg of waste/kg of products) range from 0.1 for oil refining to values higher than 25 in the pharmaceutical sector. The author favors use of the E factor metric because in an ideal process E factor is 0, whereas PMI is 1. Let us, however, underline that experimentally it is more difficult to determine the amount of waste in a process (required to calculate E factor) than the amount of entering materials (required to calculate PMI).

## 5. The EcoScale

When a more exhaustive determination of the greenness of a chemical reaction is required, many other factors than mass balances should be considered. Among them, let us mention energy balances, which include calculations of the energy used to perform the reaction itself (Joule/kg of product) but also calculations of the energy used to extract or prepare and to recycle or destruct reagents, solvents, or auxiliaries. Prices, ease and safety of handling, renewability, atmospheric emissions, and environmental risks must also be taken in account, not only in the case of the reaction itself but again in steps preceding and following it. Therefore, studies “from cradle to grave” must be conducted *i.e.*, “life cycle analyses” (LCA) [25,26,27], whose principles and framework can be found in the ISO 14040:2006 document [28]. It is not easy to perform such life cycle analyses so we would like to point out the existence of a highly simplified but indicative approach that has been developed by Van Aken *et al.* [29] in 2006. It is a semi-quantitative tool that comprises six parameters characterizing a reaction: yield, prices of the components, safety, technical set-up, temperature and time, work-up and purification. Within each of these parameters, individual penalty points are associated to particular situations, which are described in Table 2. The ideal score is attributed to an ideal reaction in which a compound A (substrate) undergoes a reaction with (or in the presence of) inexpensive compounds B to give the desired product C in 100% yield at room temperature with a minimal risk for the operator and a minimal impact for the environment. The actual EcoScale score is then calculated by lowering the ideal score of 100 by those penalty points. A value higher than 75 characterizes an excellent process; between 75 and 50, the score corresponds to an acceptable process; and below 50 the process is not acceptable. An automatic calculator is available online [30]. Many discussions can arise because of the subjective weight of the penalties and the subjective nature of the situations encountered in the process. Nevertheless, the work of Van Aken *et al.* has the merit to exist and to provide a rapid tool of evaluation, which can, moreover, be adapted by anyone. In 2012, a modified version has been proposed by Dash *et al.* [31] from Boehringer Ingelheim Pharmaceuticals company.

Penalty points and overall EcoScale scores calculated for Routes 1–3 are gathered in Table 3.

Values collected in Table 3 demonstrate that safety plays a dramatic role in the evaluation of the performances of the various protocols affording paracetamol. Amazingly, whereas the Hoechst-Celanese process emerged as a performing procedure when mass balances only were considered, that process requires as many experimental precautions as the two other synthetic routes. Temperature conditions, essentially cooling, are additional factors rendering Route 3 less environmentally friendly than expected from, for example, estimation of the atom economy.

## 6. The Radial Polygon Representation

**Figure 5:**
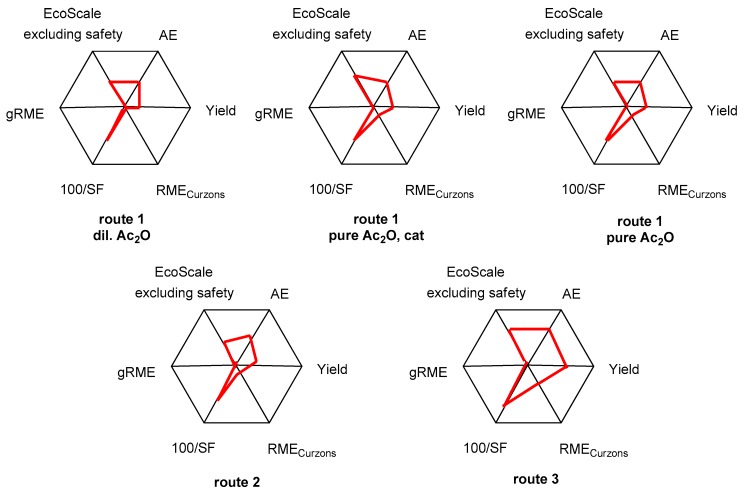
The radial hexagon representations for the preparation of paracetamol, where AE = atom economy, SF = stoichiometric factor, RME_Curzons_ = reaction mass efficiency following Curzons, and gRME = global reaction mass efficiency.

Values collected in Table 1 and Table 3 can readily be inserted in radial polygons [17,32,33] in which each summit would represent a value of 100% and the center a value of 0%; note that 100/SF rather than SF is used in those representations. Although pentagons are often preferred for that purpose, we chose the hexagon by assigning one summit to the EcoScale value (excluding the penalty points for safety). The summits of the polygon correspond to the ideal process, whereas joining the coordinates on the radius will give a distorted polygon depicting the actual procedure, as illustrated in Figure 5. Distortions will help the chemist to identify parameters that must be improved in order to perform a more efficient and consequently a more environmentally friendly synthesis. Examination of Figure 5 reveals that Route 3 constitutes a reasonable synthetic sequence when compared with Routes 1 and 2. However, reaction mass efficiency as defined by Curzons should be increased. This could be accomplished by: (i) reducing the excess of acetylating agent (two-fold excess of acetic acid); (ii) using commercially available hydroxylamine (rather than the hydrochloride salt that must be neutralized); and (iii) improving the yield of the Beckmann rearrangement step. It is noteworthy that all of the protocols we considered, including Route 3, are characterized by extremely deplorable global reaction mass efficiency and penalty points for safety. Global RME could be modified by adjusting the volumes of solvents used in the workup procedures. Safety, as defined in the EcoScale, could hardly be modified because it is determined by the nature of the reagents and all auxiliary substances involved in the reactions.

## 7. Conclusions

Because all of us are conscious that our environment must be protected and kept as clean as still possible, many efforts have been and are necessitated in order to prevent formation and accumulation of waste as well as emission of greenhouse gases. Chemists can take part in those challenges and there is no doubt that the birth of so-called “*green chemistry*” has already modified the ways researchers are conceiving chemical transformations. Actually, some quantitative tools are available to evaluate the efficiency of a reaction and to point out its weaknesses in terms of ecological, economical, and safety impacts. Some of those tools are described in this review and applied to the preparation of paracetamol following three different routes. Some chemists would claim that several individual steps constitute green reactions because, for example, they are performed in water or in the absence of solvent. However, when looking at metrics used in green chemistry, it clearly appears that the use of water as the solvent or even the absence of solvent have little influence on the overall performances of the chemical transformations yielding paracetamol.

There are numerous articles, reviews, and books dealing with green chemistry. A short and arbitrary selection can be found in references [34,35,36,37,38,39,40,41,42,43,44,45,46,47].

## Figures and Tables

**Table 1:** Metrics associated with protocols considered for the preparation of paracetamol.

	AE (%)	Yield (%)	RME_Curzons_ (%)	SF	gRME (%)
Route 1					
Nitration of phenol	50	37	10	1.79	4
Reduction	62	74	37	1.22	2
Acetylation (dil. Ac_2_O)	72	60	38	1.12	7
Overall (dil. Ac_2_O)	36	16	4	1.67	1
Acetylation (pure Ac_2_O, cat.)	72	91	23	1.92	4
Overall (pure Ac_2_O, cat.)	36	25	6	2.08	1
Acetylation (pure Ac_2_O)	72	97	69	1.01	69
Overall (pure Ac_2_O)	36	27	7	1.61	2
Route 2					
Acetylation (AcCl)	81	99	79	1.01	79
Overall (AcCl)	38	27	8	1.64	2
Route 3					
Acetylation of phenol	88	96	61	1.39	6
Oximation	63	99	58	1.09	8
Rearrangement	100	71	71	1.00	5
Overall	58	68	31	1.32	2

**Table 2:** Penalty points used in the EcoScale.

Parameters	Penalty Points
**Yield**	**(100 − Effective Yield)/2**
Price of the reaction components (to obtain 10 mmol)	
Inexpensive (< 10 US$)	0
Expensive (between 10 and 50 US$)	3
Very expensive (> 50 US$)	5
Safety (adapted for the Globally Harmonized System of Classification and Labeling of Chemicals)	
GHS09 (dangerous for the environment)	5
GHS06 (toxic)	5
GHS02 (flammable)	5
GHS01 (explosive)	10
GHS07, GHS08 (extremely toxic)	10
**Technical setup**	
Common setup	0
Instruments for controlled addition (dropping funnel, *etc*.)	1
Unconventional activation technique (microwave, *etc.*)	2
Pressure equipment > 1 atm	3
Any additional special glassware	1
(Inert) gas atmosphere	1
Glove box	3
**Temperature/Time**	
Room temperature, < 1 h	0
Room temperature, < 24 h	1
Heating < 1 h	2
Heating > 1 h	3
Cooling to 0 °C	4
Cooling < 0 °C	5
**Workup/Purification**	
None	0
Cooling to room temperature	0
Adding solvent	0
Simple filtration	0
Removal of solvent with bp < 150 °C	0
Crystallization and filtration	1
Removal of solvent with bp > 150 °C	2
Solid phase extraction	2
Distillation	3
Sublimation	3
Liquid-liquid extraction	3
Classical chromatography	10

**Table 3:** Penalty points and overall EcoScale scores for the preparation of paracetamol.

Parameters	Penalty Points for Route				
	1, Acetylation with dil. Ac_2_O	1, Acetylation with Pure Ac_2_O and Cat.	1, Acetylation with Pure Ac2O	2	3
Yield	42.0 (37%)	37.5 (25%)	36.5 (27%)	36.5 (27%)	16 (68%)
Safety					
Reagents ^a^	45	45	45	50	60
Intermediates ^b^	50	50	50	50	20
Solvents, auxiliaries ^c^	30	30	30	30	50
Technical setup	1	1	3	3	4
Temperature/Time	8	8	8	8	14
Workup/Purification	6	6	6	6	6
Overall EcoScale scores (excluding safety)	43.0	47.5	46.5	46.5	60.0

^a^ Phenol (GHS05, GHS08); sulfuric acid (GHS05); sodium nitrate (GHS07); sodium borohydride (GHS02, GHS06); acetic anhydride (GHS02, GHS06); acetyl chloride (GHS02, GHS07); acetic acid (GHS02); hydroxylamine hydrochloride (GHS07, GHS08, GHS09); NH_4_OH (GHS07, GHS09); ^b^ 4-Nitrophenol (GHS05, GHS08); 2-nitrophenol (GHS07); 4-aminophenol (GHS07, GHS08, GHS09); 4′-hydroxyacetophenone (GHS07); 4’-hydroxyacetophenone oxime (GHS07); ^c^ Pd/C (GHS07); HCl (GHS07); NaHCO_3_ (GHS07); HF (GHS06); KOH (GHS07); C_2_H_5_OH (GHS02, GHS 07, GHS 08); trifluoroacetic acid (GHS07).
